# *Rahnella aquatilis* JZ-GX1 alleviates iron deficiency chlorosis in *Cinnamomum camphora* by secreting desferrioxamine and reshaping the soil fungal community

**DOI:** 10.3389/fpls.2022.960750

**Published:** 2022-09-15

**Authors:** Wei-Liang Kong, Ya-Hui Wang, Lan-Xiang Lu, Pu-Sheng Li, Yu Zhang, Xiao-Qin Wu

**Affiliations:** ^1^Co-Innovation Center for Sustainable Forestry in Southern China, College of Forestry, Nanjing Forestry University, Nanjing, Jiangsu, China; ^2^Jiangsu Key Laboratory for Prevention and Management of Invasive Species, Nanjing Forestry University, Nanjing, Jiangsu, China

**Keywords:** *Cinnamomum camphora*, iron deficiency chlorosis, *Rahnella aquatilis*, microbial community, desferrioxamine

## Abstract

Plant growth-promoting rhizobacteria are important for improving plant iron nutrition, but the interactions among inoculants, host plants and soil microorganisms have not been greatly explored. *Rahnella aquatilis* JZ-GX1 was applied to treat the increasingly serious iron deficiency chlorosis in *Cinnamomum camphora*, and the resulting improvement in chlorosis was determined by assessing the contents of chlorophyll, active iron, Fe^2+^ and antioxidant enzymes in leaves, the effects on the soil microbial community and the metabolism in the rhizosphere by high-throughput sequencing techniques and liquid chromatography–mass spectrometry (LC–MS). The results showed that inoculation with JZ-GX1 significantly increased the chlorophyll content of *C. camphora*, which promoted the redistribution of active iron in roots and leaves, increased the activities of superoxide dismutase (SOD), peroxidase (POD), catalase (CAT) and ascorbate peroxidase (APX), and thus reduced membrane damage in iron-deficient *C. camphora* caused by reactive oxygen species. According to genome prediction and ultra-performance liquid chromatography–mass spectrometry (UPLC–MS) analysis, the JZ-GX1 strain could secrete desferrioxamine (DFO), and the concentration of DFO in *C. camphora* rhizosphere was 21-fold higher than that in uninoculated soil. The exogenous application of DFO increased the SPAD and Fe^2+^ contents in leaves. In addition, the inoculant affected the fungal community structure and composition in the *C. camphora* rhizosphere soil and increased the abundances of specific taxa, such as *Glomus*, *Mortierella*, *Trichoderma,* and *Penicillium*. Therefore, *R. aquatilis* JZ-GX1 application promoted iron absorption in *C. camphora* trees by secreting DFO and alleviated iron deficiency chlorosis through interactions with the local fungal community.

## Introduction

Iron is one of the most important trace elements in many living organisms and mainly exists as Fe^2+^ and Fe^3+^ in nature (Liu et al., 2017). The transformation between these forms is an important redox process in living cells, and the availability and content of iron play important roles in the growth and development of plants (Ferreira et al., 2019). Although iron is abundant in the Earth’s crust, ranking fourth among all elements on the Earth’s surface, the solubility of iron in soil is very low due to oxidation by oxygen in the atmosphere, drought and the alkalinity of semidry calcareous soil; thus, the absorption of iron by plants is difficult (Zhou et al., 2016). The concentration of free soluble iron in soil is <10^−17^ M, which is markedly lower than the optimal value for plant growth (Arikan et al., 2018). Plants growing in these soils often show iron deficiency chlorosis, which seriously affects the yield of crops (Aras et al., 2018). Therefore, iron deficiency stress has become a global problem. Over the years, researchers have also introduced many control measures to solve this problem, including soil application, trunk injection, and foliar spraying of various inorganic, organic and chelated iron fertilizers (Farshchi et al., 2021). However, these methods have many problems, such as causing slight damage to trees and environmental pollution and high cost; thus, no economic and effective method for the prevention and control of iron deficiency chlorosis in plants has been developed (Li M. et al., 2021).

A large number of microorganisms are found in plant rhizosphere soil, and among these, the bacteria beneficial to plants are called plant growth-promoting rhizobacteria (PGPR). Many studies have shown that PGPR can secrete a series of substances, including siderophores, organic acids, and volatiles, to help plants cope with iron deficiency stress (Radzki et al., 2013; Martinez-Medina et al., 2017). For example, Liu et al. (2017) effectively controlled the yellowing associated with iron deficiency in peanuts and peaches through the use of siderophore-producing bacteria. Aras et al. (2018) studied the effect of PGPR on the uptake of iron by peach trees in calcareous soil and found that the absorption and transport of malic acid released by bacteria in the rhizosphere effectively reduced the pH of leaves and that the organic acid secreted by *Azospirillum brasilense* significantly reduced the pH of cucumber hydroponic nutrient solution and increased the activity of Fe^3+^ reductase in cucumber roots. However, the mechanisms through which microorganisms promote plant iron absorption are currently mostly limited to the phenotypic and physiological responses of host plants, and few studies have investigated the changes in indigenous soil microbial communities after inoculation with exogenous bacterial agents. The effects of most microorganisms on plants in nature are accomplished through interactions (Niu et al., 2021). Due to the influence of biological and environmental factors, the effects of inocula are often not ideal. The interaction between the plant rhizosphere and microorganisms is very complex. Whether a single exogenous bacterial agent can adapt to the indigenous microbial community and exert its plant growth-promoting characteristics is currently unclear. Therefore, understanding the interactions among plants, inocula and local microorganisms is of great significance.

In recent years, with the rise and development of genomics technology, an increasing number of studies have used genomic, transcriptome, metabolome and microbiome sequencing techniques to comprehensively and deeply study the interaction mechanisms between PGPR and plants or the soil microbial community (Gamez et al., 2019; Luo et al., 2020; Yang et al., 2020; Ye et al., 2020; Priya et al., 2021). For example, Li F. et al. (2020) combined transcriptome and microbiome analyses and revealed that the rare fungus *Mortierella capitata* can promote crop growth directly by altering root gene expression levels and indirectly *via* interaction with indigenous rhizosphere bacteria. Tao et al. (2020) combined high-throughput sequencing, culturable microbiome, key microbial return and community interaction experiments and found that *Bacillus amyloliquefaciens* W19 enhanced multispecies root colonization in coordination with specific probiotic *Pseudomonas* populations in the tomato rhizosphere in the treatment of tomato bacterial wilt to ultimately result in the formation of a probiotic community that effectively helps plants resist pathogen infection. Tsai and Schmidt (2017) showed that coumarin secreted by plants is favorable for the interactions between plants and microbial communities under iron restriction. These special metabolites change the composition of root microorganisms and are necessary for plant iron uptake and immune regulation mediated by microbial communities.

*Cinnamomum camphora*, which belongs to Lauraceae, is the main component of subtropical evergreen broad-leaved forests and a famous tree species in landscapes (Zhong et al., 2019). Due to its good appreciation, pruning resistance, strong sprouting ability and excellent cultural value, *C. camphora* has become one of the dominant tree species in landscaping, has been widely used in parks, green spaces, street trees and courtyard greening (Chen et al., 2020) and has played an important role in improving the ecological environment of cities. *Cinnamomum camphora* contains unique chemicals and is affected by few diseases and insect pests, but due to heavy planting, lack of management and the area not suitable for the growth of the species, *C. camphora* etiolation is becoming increasingly serious in many places (Zhou H. et al., 2017; Duan et al., 2020). *Cinnamomum camphora* iron deficiency etiolation is generally characterized by varying degrees of leaf yellowing, poor tree potential, white and thin leaves in severe cases, scorched and withered spots on the tip and edge of leaves, and vulnerability to frost injury; the yellowed leaves are initially locally distributed only on the crown, and the number then increases gradually, resulting in yellowing of the whole crown, withering of shoots, sparseness of branches and leaves, shrinking of the crown, gradual exhaustion and death, which seriously affects the economic and ecological benefits of the species (Kong et al., 2020a).

The etiolation of *C. camphora* is pervasive and widespread. Researchers usually explore the cause of the disease from the perspective of the soil pH and element content, whereas the microbial community in the underground part of *C. camphora* has rarely been investigated. In this study, nontargeted metabolic technology based on LC–MS was used to characterize the secretion and metabolism of *Rahnella aquatilis* JZ-GX1 in the rhizosphere of *C. camphora* and to identify the key substances that may affect the uptake of iron by *C. camphora*. From the point of view of soil microecology, we aimed to clarify the mechanism through which *R. aquatilis* JZ-GX1 alleviates the etiolation of *C. camphora*. A microbial amplification technique was also used to explore the effect of the JZ-GX1 strain on the community structure and composition of bacteria and fungi in the rhizosphere of *C. camphora* and to explore the soil microbial inducement that may lead to the etiolation of *C. camphora* with the aim of providing a scientific solution for the management of the disease.

## Materials and methods

### Plant materials, growth conditions and pot experimental design

*Rahnella aquatilis* JZ-GX1 (CCTCC M2012439) was isolated from the rhizosphere soils of *Pinus massoniana* in Guangxi, China, and identified by 16S rRNA sequencing (GenBank No. KC351183.1). This bacterial strain was inoculated into Luria-Bertani (LB) liquid medium and incubated in an orbital shaker (200 rpm) at 28°C for 18 h. Bacteria were collected by centrifugation at 8,000 rpm and 4°C for 15 min. The centrifuge tubes were washed with 0.1 M phosphate-buffered saline (PBS, pH 7.2), and the bacteria were then diluted to obtain 1 × 10^7^ CFUs/ml for microbial inoculation (Kong et al., 2020b).

Three-year-old *C. camphora* seedlings were purchased from a nursery and planted in plastic pots (Ф15 × 20 cm) containing 2 kg of saline-alkali soil. The soil properties were as follows: pH 8.2; organic matter, 7.89 g/kg; available N, 37.61 mg/kg; available P, 16.07 mg/kg; available K, 153.01 mg/kg and effective Fe, 6.14 mg/kg. All the plants were cultured at 25°C under a 12-h light/12-h dark cycle until the seedlings started to exhibit yellowing. Subsequently, 60 *C. camphora* seedlings were selected, divided into three groups (20 plants per group) and administered one of the following treatments: (1) 0.1 M PBS, (2) 1.2% iron sulfate (FeSO_4_), and (3) 10^7^ CFUs/mL *R. aquatilis* JZ-GX1 suspended in PBS. The plant roots in each pot were inoculated with 50 ml of the treatment solution, and the plants were then watered daily until harvesting of the plant tissues. The harvested samples were used for various physiological and biochemical analyses.

### Determination of the Chl and Fe concentrations in *Cinnamomum camphora* plants

After 30 days, the concentrations of Chl in the leaves were determined according to the method described by Zhang et al. (2009). Fresh leaf tissue (0.5 g) was extracted with 2 ml of 95% (v/v) ethanol for 24 h in the dark. The concentrations of Chl a, Chl b, and carotenoids in the extract were determined by reading the absorbances at 665, 649, and 470 nm, respectively, with a spectrophotometer (UNICOWFUV-2000, Unico, United States).

After harvest, the plants were separated into leaves, shoots and roots. Approximately 0.2 g of plant material was individually digested in 1 mol/L HCl at a 1:10 ratio (v/v) for the extraction of active Fe (Koseoglu and Acikgoz, 1995). Improved NH_4_F masking method was used for the extraction of Fe^2+^ from the fresh samples. The concentrations of active Fe and Fe^2+^ in the digested solution were then determined with O-phenanthroline spectrophotometry (Ni et al., 2015).

### Determination of the MDA, EL, O_2_^−^, and H_2_O_2_ levels in leaves

To analyse the mitigation of microbe-mediated iron deficiency stress in planted soil, the stress-related levels of malonaldehyde (MDA) and reactive oxygen species (ROS) in leaves were investigated (Chen et al., 2015). Young leaves on the top of the *C. camphora* trees were collected after 3, 5, 10, and 20 days of the different treatments. Malonaldehyde was extracted from leaves with 10% trichloroacetic acid, and its concentration was determined based on the absorbances at 450, 532, and 600 nm (Xu et al., 2008). The formation rate of O_2_^−^ (Elstner and Heupel, 1976) and the level of H_2_O_2_ (Mukherjee and Choudhuri, 1983) in leaves were determined based on the absorbances at 530 and 436 nm, respectively. The values of electrolyte leakage (EL) were determined according to the method reported by Jiang and Zhang (2001).

### Determination of antioxidant enzyme activity in leaves

To verify the levels of O_2_^−^ and H_2_O_2_, ROS-scavenging antioxidant enzymes were extracted from leaves according to methods described by Liu et al. (2010). The activities of superoxide dismutase (SOD), catalase (CAT), peroxidase (POD) and ascorbate peroxidase (APX) were determined based on the absorbances at 560, 240, 470, and 340 nm, respectively (Beauchamp and Fridovich, 1971; Aebi, 1984; Zhu et al., 2004; Li J. et al., 2020). To calculate the activities of antioxidant enzymes, the protein content in each of the enzyme extracts was determined based on the absorbance at 595 nm (Bradford, 1976).

### Prediction of secondary metabolites of *Rahnella aquatilis* JZ-GX1

The complete genome sequence of *R. aquatilis* JZ-GX1 has been deposited in the NCBI under GenBank accession number SAMN18652319, and the secondary metabolites of the JZ-GX1 strain were predicted using antiSMASH online prediction software.^1^ The complete gene cluster information for siderophore synthesis in the JZ-GX1 genome was determined and mapped. The secondary metabolites of JZ-GX1 and other bacteria were compared and analyzed (Blin et al., 2019).

### Gene expression related to siderophore biosynthesis of JZ-GX1 under Fe deficiency

Strain JZ-GX1 was inoculated into 20 ml of MSA medium (per liter: 20 g of sucrose, 2 g of L-asparagine, 1 g of K_2_HPO_4_, 0.5 g of MgSO_4_·7H_2_O) with or without 20 μM FeCl_3_·6H_2_O for 24 h and centrifuged at 12,000 rpm for 5 min. The bacterial cells were collected, rinsed with sterile water, frozen in liquid nitrogen, and stored at −80°C for RNA isolation. Total RNA of JZ-GX1 was extracted using a bacterial RNA extraction kit (Jiancheng Bioengineering Institute, Nanjing, China). After the detection of RNA quality and concentration, cDNA samples were prepared using HiScript II Q Select RT SuperMix for RT–qPCR (CAT: 11202ES08; Yeasen, Shanghai, China). The expression levels of related genes were calculated using ABI 7500 software (Applied Biosystems, United States), and *atpD* was used as an internal control (Li P. et al., 2021). These gene primers are shown in Supplementary Table S1.

### Ultra-performance liquid chromatography–mass spectrometry spectrometric analysis

The crude siderophore extract of *R. aquatilis* JZ-GX1 was syringe-filtered using a 0.22-μm nylon filter. Filtrates were injected into a UHPLC instrument (U3000, Thermo Scientific, Germany) with a Wates BEH C18 Column (150 × 2.1 mm, 1.7 μm, United States) at 35°C, and the instrument was connected to a mass spectrometer (TripleTOF^®^ 5600+, AB Sciex, United States). The mobile phase consisted of acetonitrile:0.1% formic acid-H_2_O at a ratio of 5%:95% with a 0.3 ml/min flow rate.

The positive and negative ion modes of electrospray ionization (ESI) were detected. The source conditions of ESI were as follows: Ion Source Gas 1 (Gas1): 50 charge; Ion Source Gas 2 (Gas2): 50 magical; Curtain Gas (CUR): 25; Source Temperature: 500°C (positive ion) and 450°C (negative ion); Ion Sapary Voltage Floating (ISVF): 5,500 V (positive ion) and 4,500 V (negative ion); TOF MS scan range: 100–1,200 Dajia; ion scan range product ion scan range: 50–1,000 Da; MaOF 0.2 s; MS scan accumulation time: 0.01 s; secondary mass spectrometry was performed by information-dependent acquisition (IDA). Using high sensitivity mode, declustering potential (DP): ±60 V, collision energy: 35 ± 15 eV.

### Identification of metabolites in the rhizosphere soil of *Cinnamomum camphora*

Rhizosphere soil of *C. camphora* with and without inoculation with the JZ-GX1 strain was extracted in prechilled methanol (3 ml), sonicated for 30 min and centrifuged for 20 min at 4,000×*g*. The supernatant was passed through a 0.22-μm filter for liquid chromatography combined with mass spectrometry (LC–MS) analysis at Wenxin Biotechnology Co., Ltd. (Nanjing, China; Wan et al., 2018). In brief, the extracts were separated using an ultra-performance liquid chromatography (UPLC) system (Waters Corporation, Japan). The UPLC column was an ACQUITY UPLC BEH C18 column (100 mm × 2.1 mm, 1.7 μm), which was maintained at 35°C and treated with a multistep gradient for product elution over the course of 30 min at 0.4 ml/min. The gradient was composed of A (formic acid-water, containing 0.1% formic acid) and B (formic acid-acetonitrile, containing 0.1% formic acid) as follows: 0–5 min, 5% B; 5–50 min, linear gradient from 5% to 100% B; 50–60 min, 100% B. The time-of-flight mass spectrometry (TOF-MS) parameters were as follows: positive and negative ionization modes; the ion source temperature and solvent removal temperature were 120°C and 450°C, respectively; the He carrier gas was 800 L/h; the MS scanning range was 50–1,000 MHz/z; and the resolution was 30,000 (Yang et al., 2020).

Before the identification of metabolites, the original data were analyzed by peak identification, alignment, deconvolution and normalization. Following data evaluation, including quality control and principal component analysis (PCA) analysis, OPLS-DA, a supervised multivariate method, was used to maximize metabolome differences between sample pairs. Differentially accumulated metabolites (DAMs) were set at fold change (FC) > 2 or FC < 0.5 and OPLS-DA VIP ≥ 1. Volcano map construction and unsupervised PCA clustering of the identified metabolites were performed using Ezinfo 3.0. The MetaboAnalyst 4.0 software was employed to build heatmap diagrams. Subsequently, the KEGG^2^ were used to construct the metabolic pathway.

### High-throughput sequencing and analysis of 16S rRNA and internal transcribed spacer regions

To investigate the effects of strain JZ-GX1 on the rhizosphere microbial community, six samples were randomly selected on the 30th day from three replicates of the inoculated and uninoculated treatments, and two soils from the same replicate were mixed to obtain one composite sample. After removing 0–5 cm of topsoil, the soil around the root system was gently shaken off. The soil attached to the root surface was then evaluated as rhizosphere soil. The fresh soil samples were stored at −80°C before DNA extraction and microbial community analysis. Soil DNA was extracted from 0.60 g of fresh soil using a Fast DNA^®^ Spin Kit for Soil (MP Biomedicals, CA, United States). Two universal primer sets (515F/909R and ITS1F/ITS2) were used: the former was used to amplify the prokaryotic 16S rRNA V4 region, and the latter was used to amplify the fungal internal transcribed spacer (ITS) region (Blaalid et al., 2013). The data were analyzed with the free online platform of the Majorbio Cloud Platform,^3^ and the obtained prokaryotic and fungal sequences were deposited in the Sequence Read Archive (SRA) with the accession number PRJNA733870.

### Estimation of root fungal colonization frequency

The 1-cm long root segments were cleared with 10% KOH solution for 100 min at 95°C, bleached with 10% hydrogen peroxide for 15 min, acidified with 0.2 mol/L hydrochloric acid for 1 h, and finally stained with 0.05% (w/v) trypan blue in lactophenol for 3 min. After microscopic observation (Zeiss Microscope System Standard 16; Carl Zeiss Ltd.; Germany), the AMF colonization rate was expressed as the percentage of the number of fungal colonized root segments over the total number of observed root segments (Yang et al., 2021).

### Data analysis and processing

The data were subjected to analysis of variance (ANOVA) followed by Duncan’s multiple comparison test with SPSS 21.0 software (IBM Inc., Armonk, NY, United States), or independent samples t-test to determine significant differences (*p* < 0.05). Graphs were generated using GraphPad Prism 8.0 (GraphPad Software, Inc., United States).

## Results

### *Rahnella aquatilis* JZ-GX1 alleviated iron deficiency chlorosis in *Cinnamomum camphora*

**Figure 1:**
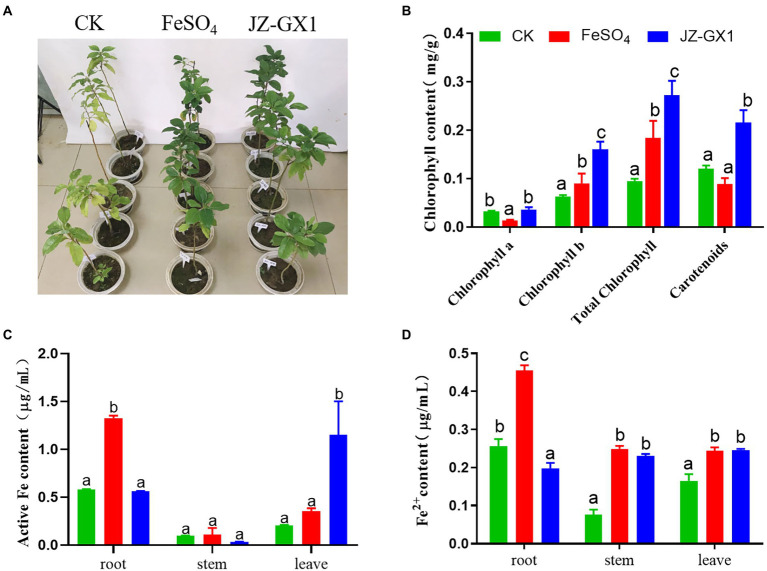
Effects of *Rahnella aquatilis* JZ-GX1 on the phenotype **(A)**, chlorophyll content **(B)**, active iron content **(C)** and Fe^2+^ content **(D)** of *Cinnamomum camphora*. The data were analyzed by one-way ANOVA followed by Duncan’s post-hoc test. Different letters indicate statistically significant differences (*p* < 0.05) among treatments.

Chlorophyll is one of the most important pigments related to photosynthesis and is widely used to evaluate the status of iron stress in plants. The application of JZ-GX1 strain significantly improved the etiolation of *C. camphora* resulting from iron deficiency (Figure 1A). Compared with CK, FeSO_4_ and inoculation treatments significantly increased the chlorophyll b, total chlorophyll and carotenoid contents of iron-deficient *C. camphora*. Among these treatments, the inoculation treatment exerted the best effect, as observed by 154.03%, 186.64%, and 80.07% increases in these contents, respectively, but had little effect on the content of chlorophyll a (Figure 1B).

Active iron is a form of iron directly absorbed and utilized by plants that can more effectively affect the iron nutritional status of plants. The iron content of *C. camphora* seedlings grown in alkaline soil was significantly lower. The FeSO_4_ treatment significantly increased the content of active iron in roots, whereas the JZ-GX1 treatment significantly increased the content of active iron in leaves, but the two treatments exerted no significant effect on active iron in stems (Figure 1C). The supply of exogenous inorganic iron sources and microbial inoculant not only increased the content of active iron in *C. camphora* but also affected the content of Fe^2+^. In contrast to the active iron, the JZ-GX1 treatment did not increase the content of Fe^2+^ in roots but significantly increased the content of Fe^2+^ in stems and leaves of *C. camphora* (Figure 1D), which indicated that the JZ-GX1 strain promoted the transfer of Fe^2+^ from roots to leaves.

### *Rahnella aquatilis* JZ-GX1 reduced oxidative damage in *Cinnamomum camphora* leaves

**Figure 2:**
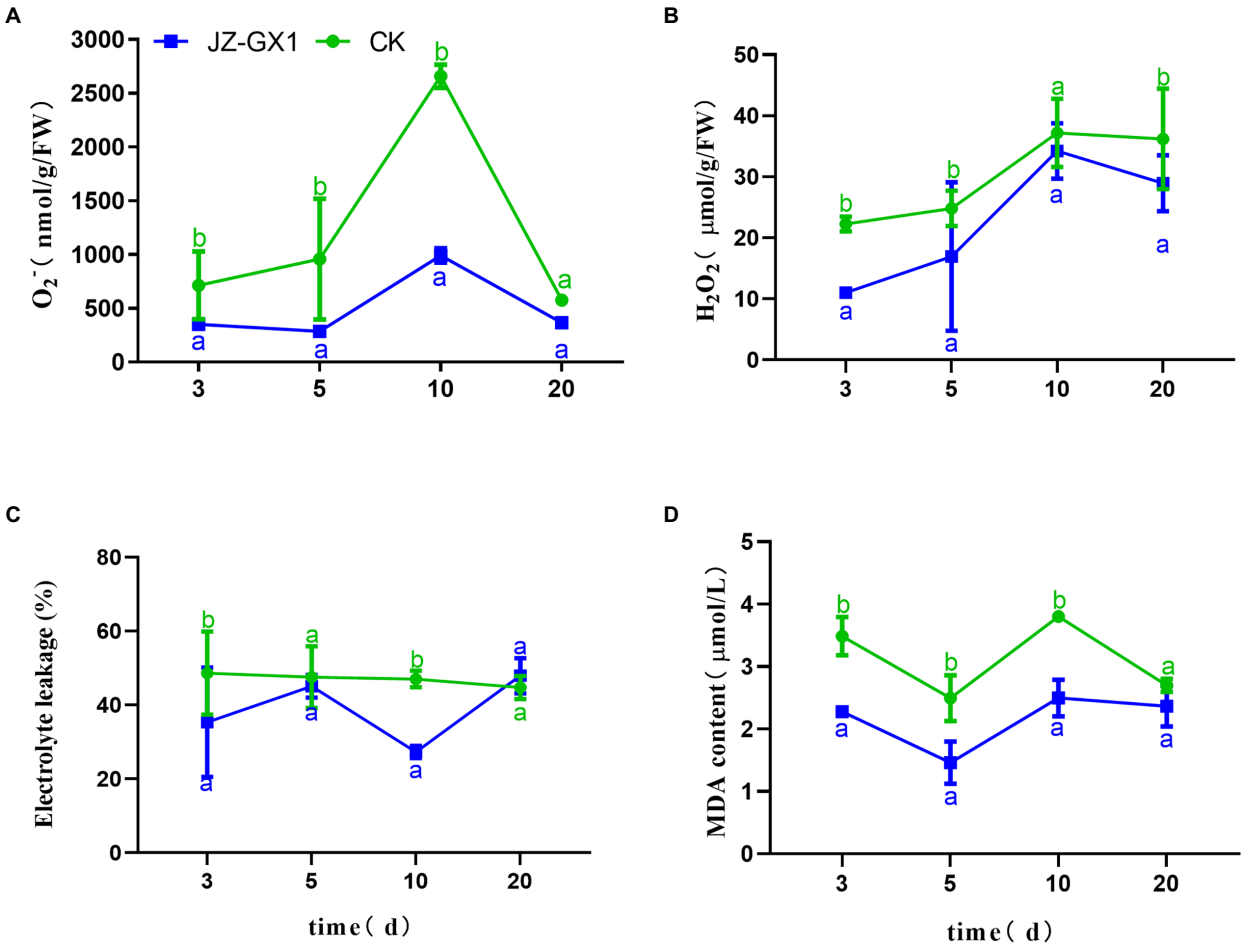
Effects of *Rahnella aquatilis* JZ-GX1 on the O_2_^−^ **(A)**, H_2_O_2_ **(B)**, EL **(C)** and MDA levels **(D)** in *Cinnamomum camphora* leaves. Data are the means ± SE. Different letters indicate statistically significant differences (*p* < 0.05) among treatments (*t*-test).

Under normal circumstances, the production and scavenging of ROS in plants are in a dynamic equilibrium, but iron deficiency inhibits the antioxidant system and increases the production of ROS, and these effects damage electron transport in chloroplasts and mitochondria and consequently cause oxidative stress in plants. The level of ROS in *C. camphora* leaves increased gradually, peaked on the 10th day, and then decreased, and the JZ-GX1 treatment decreased the contents of O_2_^−^ and H_2_O_2_ (Figures 2A,B). Electrolyte leakage analysis showed that the CK group exhibited relatively stable EL during the detection period, whereas the JZ-GX1 treatment showed some fluctuations, but the value obtained with the JZ-GX1 treatment was generally lower than that of CK (Figure 2C). Malonaldehyde is the final product of membrane lipid peroxidation in plant cells. Compared with CK, the addition of JZ-GX1 strain significantly decreased the MDA content in leaves at all tested time points with the exception of 20 days (Figure 2D).

### *Rahnella aquatilis* JZ-GX1 enhanced the activities of antioxidant enzymes in *Cinnamomum camphora* leaves

**Figure 3:**
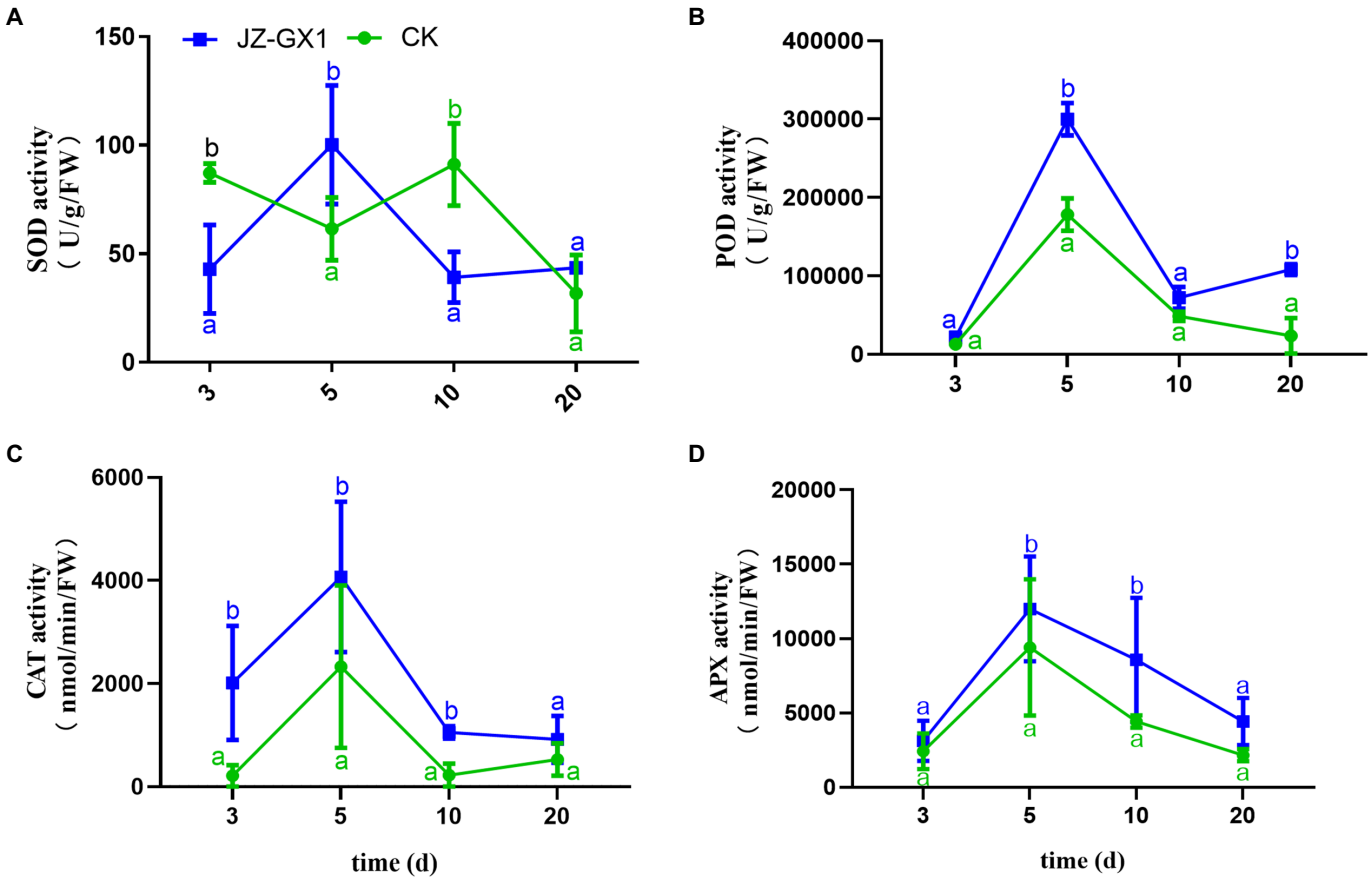
Effects of *Rahnella aquatilis* JZ-GX1 on SOD **(A)**, POD **(B)**, CAT **(C)** and APX enzyme activities **(D)** in *Cinnamomum camphora* leaves. Data are the means ± SE. Different letters indicate statistically significant differences (*p* < 0.05) among treatments (*t*-test).

The exposure of plants to external environmental stress leads to the production of ROS and subsequent oxidative damage. The scavenging of ROS in plants is mainly performed by some enzyme systems and antioxidants, and SOD, POD, CAT, and APX are important ROS-scavenging enzymes in plants. In this study, the effect of inoculation of JZ-GX1 strain on SOD enzyme activity was not obvious, only higher than that of CK on the 5th day, but lower than that of CK at other time points (Figure 3A). After the application of JZ-GX1, the activities of POD, CAT, and APX increased during culture, reached maximal levels on the 5th day, were 40.57%, 42.75%, and 21.63% higher than those obtained with the CK, respectively, and then decreased gradually but remained higher than those in the CK (Figures 3B–D).

### *Rahnella aquatilis* JZ-GX1 secreted desferrioxamine

**Figure 4:**
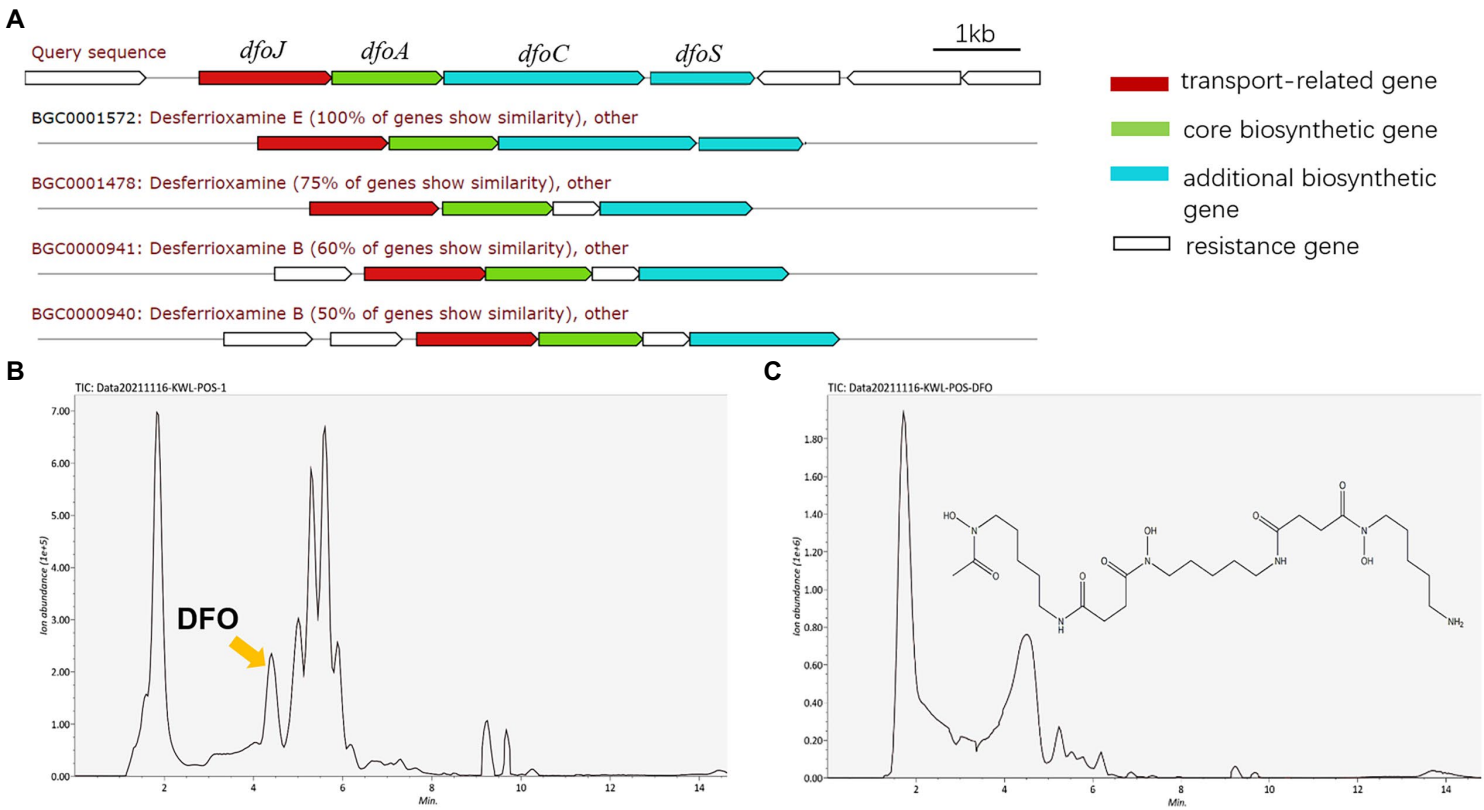
DFO, one of the predicted secondary metabolites of *Rahnella aquatilis* JZ-GX1. Biosynthetic gene cluster for DFO **(A)**, HPLC chromatogram of siderophores from JZ-GX1 culture **(B)** and DFO standard in the positive ion mode **(C)**.

To explore the potential key substances of JZ-GX1 that promote iron uptake by *C. camphora*, we predicted its secondary metabolites according to its whole genome. The results showed that JZ-GX1 strain could synthesize and secrete siderophores. The comparison found that the siderophore synthesis gene cluster of JZ-GX1 strain exhibited high homology (100%) with the known DFO E synthesis gene cluster dfoJACS of *Pantoea agglomerans* (Figure 4A). In addition, at transcription level, Fe deficiency induced the upregulation of *dfoJ*, *dfoA*, *dfoC*, and *dfoS* genes in JZ-GX1 (Supplementary Figure S1).

Moreover, the supernatant organic extracts of strain JZ-GX1 were analyzed by UPLC–MS in the positive ion mode, and the mass spectrum showed a molecular ion peak at m/z 561.3599 ([M + H]^+^), with a retention time of 4.19 min (Figure 4B), which was similar to that of the standard DFO (m/z 561.3000 ([M + H]^+^), retention time of 4.34 min; Figure 4C). Based on the mass spectra, the compound was also identified as DFO (Supplementary Figure S2).

### Application of *Rahnella aquatilis* JZ-GX1 changed the metabolite profiles in the rhizosphere soil of *Cinnamomum camphora*

**Figure 5:**
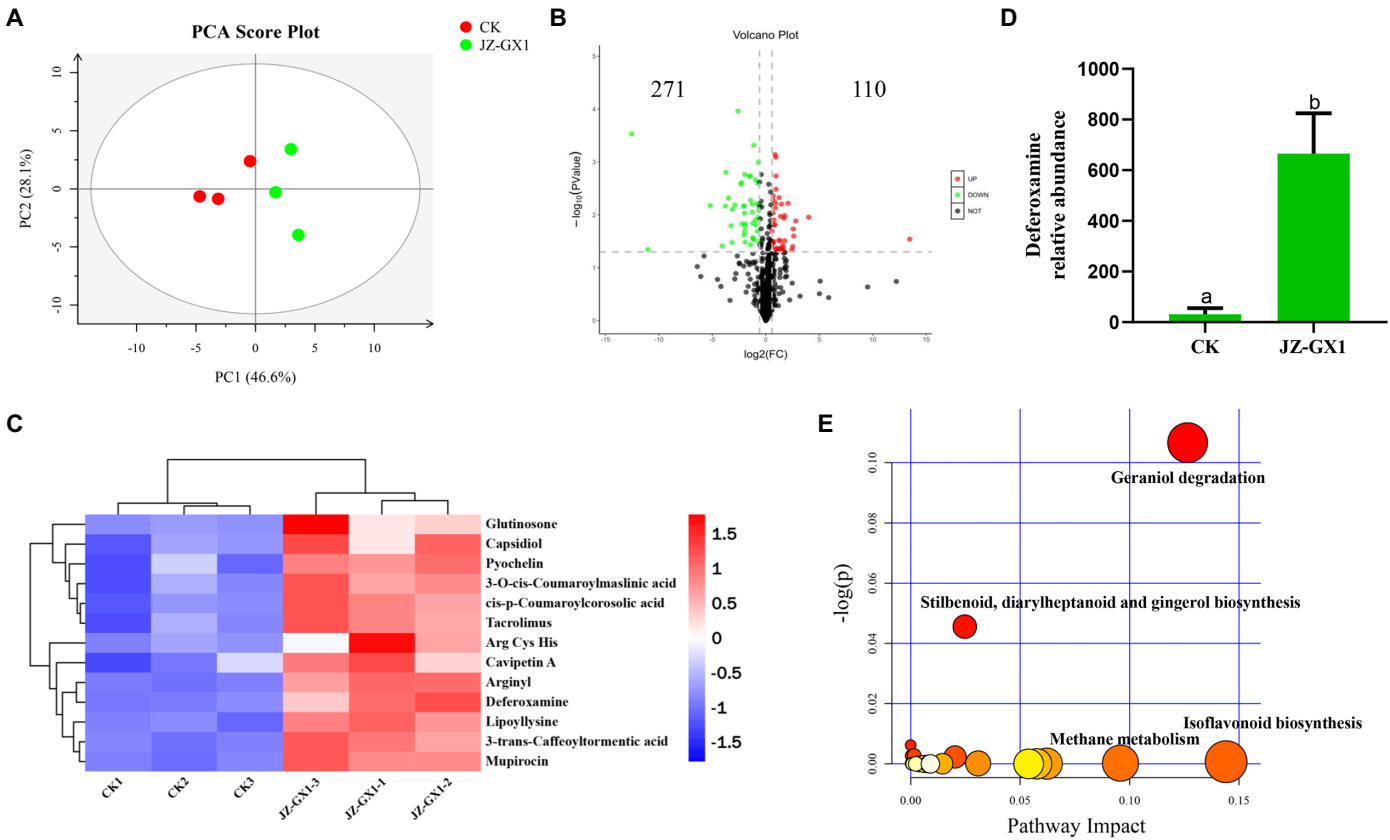
PCA analysis of the metabolites for positive mode. PC1 and PC2 are the first two principal components **(A)**, volcano plot **(B)**, heatmap analysis for the identified metabolites with significant difference being selected by PLS-DA (VIP >1, *p* < 0.05) **(C)**, DFO relative abundance **(D)** and KEGG pathway analysis of the rhizosphere soil in etiolated and green *Cinnamomum camphora.* The size and color of each point represents the number of metabolites enriched in a particular pathway and the -log_10_ *p-*values, respectively. A larger pathway impact and −log_10_ *p*-values shows a greater degree of enrichment **(E)**.

To evaluate the metabolic changes in the secretions from *C. camphora* roots induced by the JZ-GX1 strain, a nontargeted metabolomics detection of rhizosphere soils from etiolated and green *C. camphora* was performed based on UPLC–MS. A PCA analysis explained the metabolites of the two treatments were obviously separated on PC1, and the contribution rate of PC1 was 46.6%, the contribution rate of PC2 was 28.1%. The results from the same group of samples were concentrated, which indicated good repeatability between groups (Figure 5A). As seen in the volcano plot, a total of 381 metabolites were changed by the JZ-GX1 treatment, including 271 with increased expression and 110 with decreased expression (Figure 5B).

The main differential metabolites included phenolic acids, such as 3-O-cis-coumaroylmaslinic acid and 3-trans-caffeoyltormentic acid; amino acids, such as Arg, Cys, and His; glutinosone; tacrolimus; and other substances, such as lipoyllysine, arginyl and DFO (Figure 5C). Among the representative compounds showing significant differences, DFO exhibited 21.53-fold higher abundance in the JZ-GX1-inoculated group than in the uninoculated group (Figure 5D). According to the relatively large changes in metabolites, pathway enrichment analysis showed that the affected KEGG metabolic pathways mainly included glycolysis/gluconeogenesis, biosynthesis of isoflavonoids and phenylpropanoids, porphyrin and chlorophyll metabolism, tryptophan metabolism and biosynthesis of antibiotics (Figure 5E).

### Desferrioxamine promoted iron absorption by *Cinnamomum camphora*

**Figure 6:**
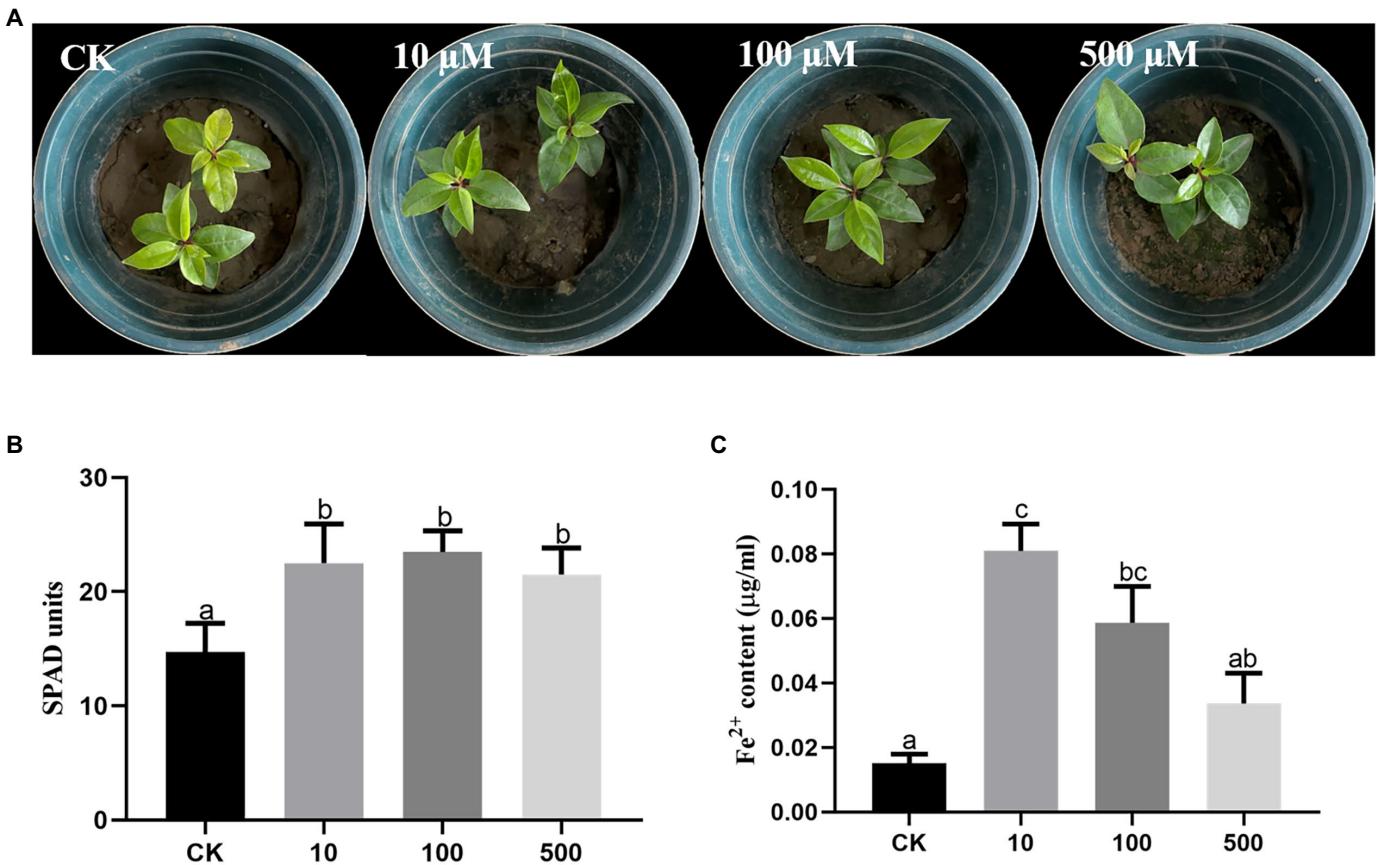
Effects of different concentrations of DFO on the growth of *Cinnamomum camphora* under iron deficiency. Plant phenotype **(A)**, SPAD **(B)** and **(C)** Fe^2+^ content. The data were analyzed by one-way ANOVA followed by Duncan’s post-hoc test. Different letters indicate statistically significant differences (*p* < 0.05) among treatments.

To explore the effects of DFO on iron absorption by *C. camphora* trees, three different concentrations of DFO were applied. The results revealed that compared with the treatment without DFO, different concentrations of DFO alleviated the iron deficiency-induced yellowing of *C. camphora* (Figure 6A). Desferrioxamine concentrations of 10, 100, and 500 μM significantly increased the SPAD value in leaves (Figure 6B). The Fe^2+^ content treated with a low concentration of DFO (10 μM) was significantly higher than that obtained with the two other concentrations (Figure 6C).

### Effects of *Rahnella aquatilis* JZ-GX1 on microbial community composition and structure in rhizosphere of *Cinnamomum camphora*

**Figure 7:**
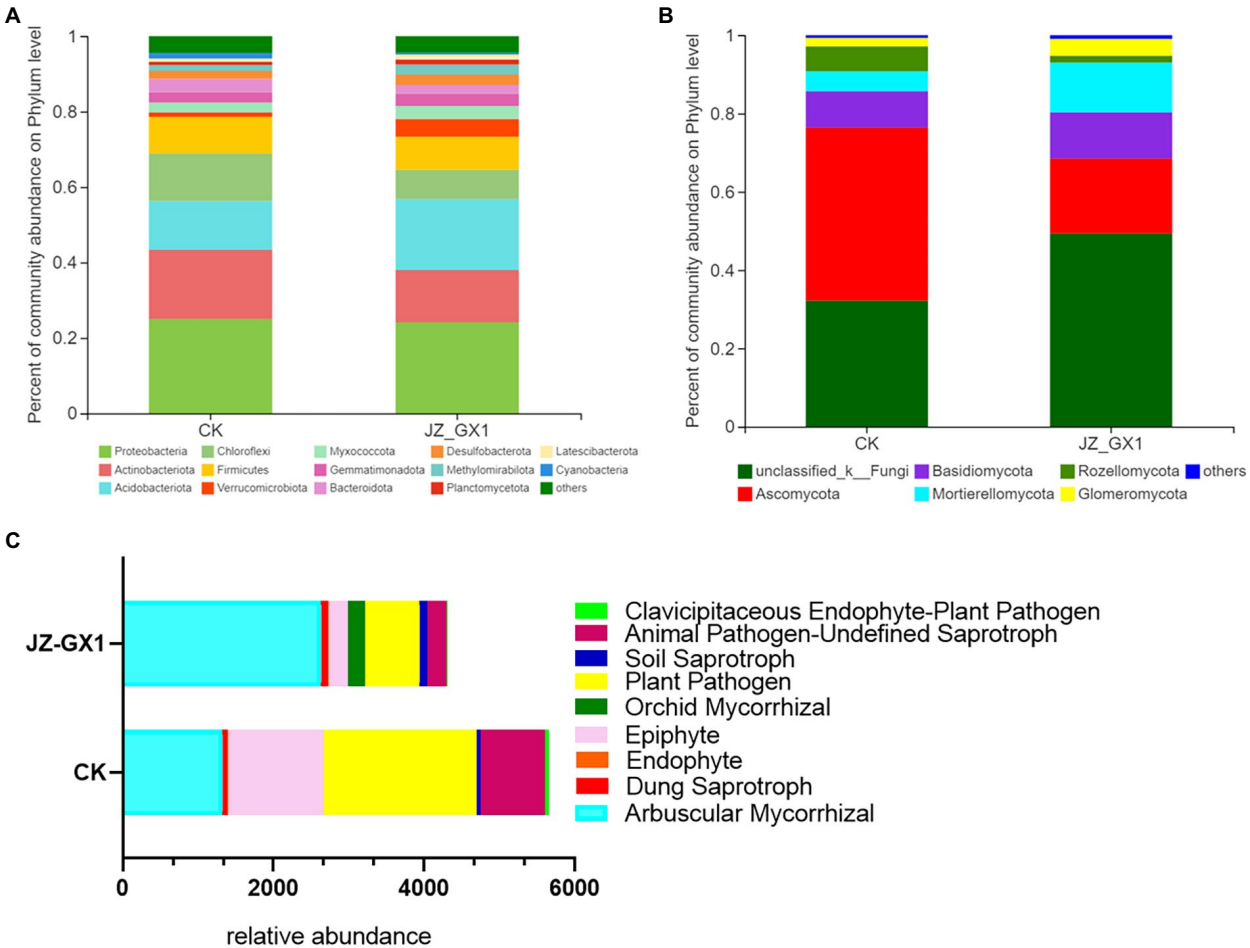
Composition and relative abundances of microorganisms in etiolated and green *Cinnamomum camphora* rhizosphere soils at the phylum level. Bacterial community structure **(A)**, fungal community structure **(B)** and FUNGuild functional prediction **(C)**.

**Figure 8:**
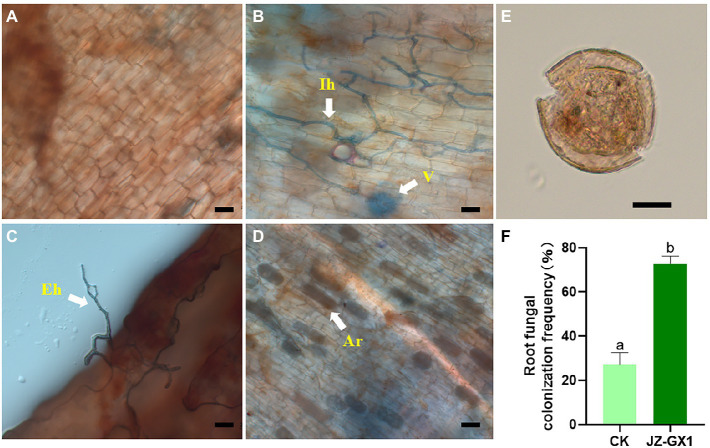
Typical structure of arbuscular mycorrhizal fungi (AMF) in the root system of *Cinnamomum camphora*. Uninfected **(A)** and infected **(B–D)** plant roots, spore morphotypes detected in the rhizosphere **(E)** and changes in root fungal colonization frequency **(F)**. Ar, arbuscule; V, vesicle; Eh, extraradical hypha; Ih, intraradical hypha. Scale bar = 20 μm.

The operational taxonomic unit (OTU) number analysis revealed a decrease in rhizosphere bacterial and fungal diversity on inoculation treatment, and the α-diversity was not affected by JZ-GX1 strain inoculation (Table 1). Regarding microbial community composition, the application increased the relative abundances of *Acidobacteriota* and *Verrucomicrobia*, slightly decreased the abundances of *Chloroflexi* and *Cyanobacteria*, and did not affect other phyla (Figure 7A). The analysis of fungal flora showed that application of the inoculum increased the relative abundances of *Basidiomycota*, *Mortierellomycota* and *Glomeromycota*, decreased the relative abundances of *Ascomycota* and *Rozellomycota*, and greatly increased the number of unclassified fungi in the rhizosphere soil of iron-deficient *C. camphora* (Figure 7B). According to the fungi functional guild (FUNGuild) prediction, the JZ-GX1 treatment also decreased the relative abundances of saprotrophic fungi, plant pathogens and animal pathogens and increased the proportions of arbuscular mycorrhizal fungi and orchid mycorrhizal (Figure 7C). The root system of *C. camphora* inoculated with JZ-GX1 formed a typical mycorrhizal structure, and a large number of arbuscules and vesicles were observed (Figures 8B–E), while the roots of most uninoculated plants were not infected by mycorrhiza (Figure 8A). The hypha colonization rate increased by 270.37% compared to CK (Figure 8F). These results indicated that the application of the JZ-GX1 strain greatly disturbed the community structure of the rhizosphere fungi of *C. camphora*.

**Table 1:** Microbial α-diversity in *Rahnella aquatilis* JZ-GX1 inoculation and control treatments.

		OTU number	Shannon	Simpson	Ace	Chao1
Bacteria	Control	888^1^	6.7180 ± 0.12a	0.0046 ± 0.001a	3703.05 ± 172.72a	3692.35 ± 132.57a
	Inoculation	804^1^	6.5103 ± 0.25a	0.0058 ± 0.002a	3651.81 ± 276.79a	3632.12 ± 278.82a
Fungal	Control	190^1^	3.9162 ± 0.24a	0.0746 ± 0.02a	450.14 ± 96.34a	450.68 ± 97.19a
	Inoculation	84^1^	3.4807 ± 0.74a	0.1209 ± 0.11a	308.18 ± 72.26a	308.69 ± 72.30a

Means ± standard deviations. Same letters indicated no significant difference between control and inoculation treatments based on Student’s *t*-test (*p* < 0.05).

1 Represents the OUT number.

### Identification of specific fungal genera

**Figure 9:**
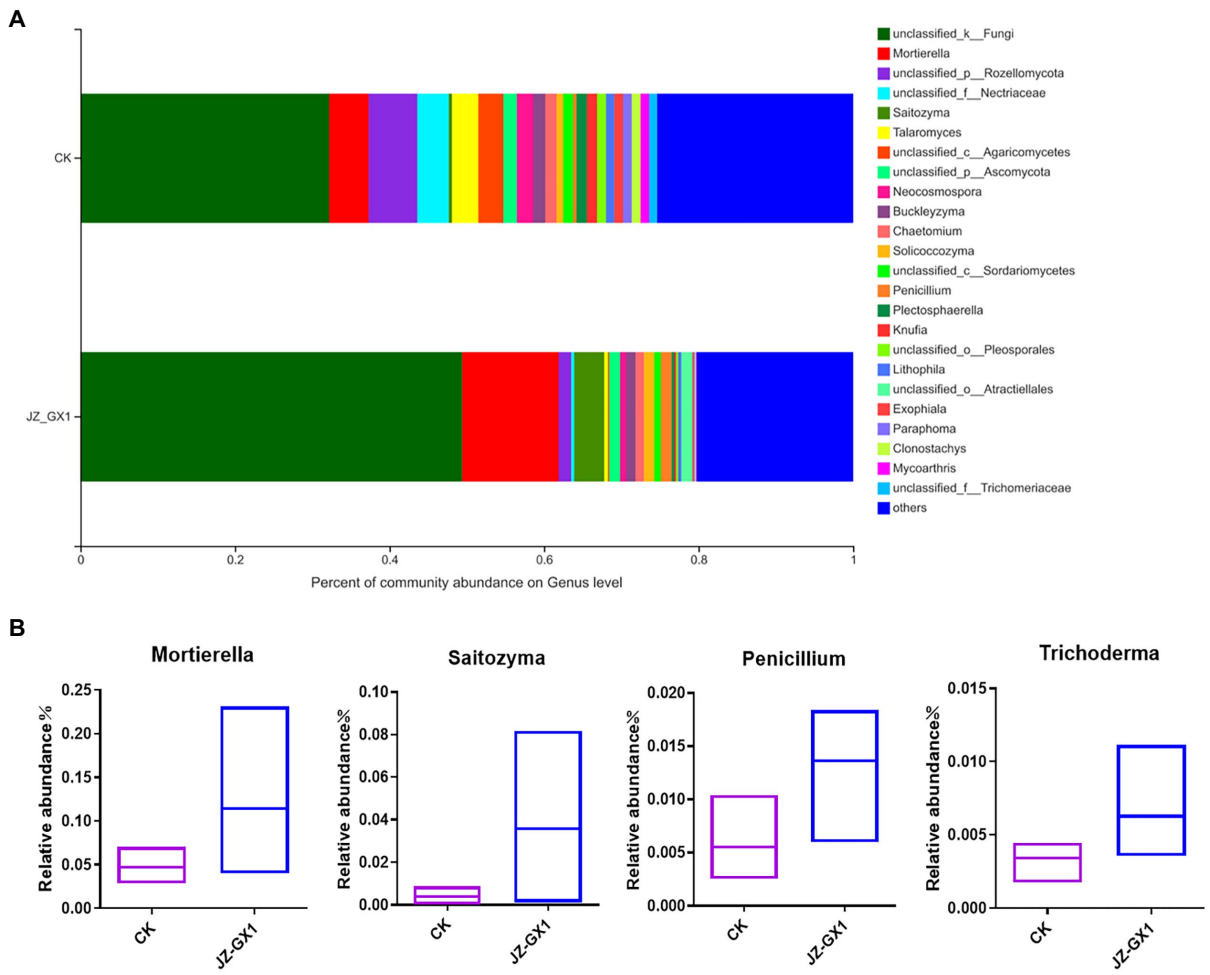
Composition of fungal communities in etiolated and green *Cinnamomum camphora* rhizosphere soils at the genus level. Proportion of fungi in each genus **(A)** and specific microorganisms **(B)**.

The histogram presented in Figure 9A showed the composition of the fungal community in the rhizosphere soils of etiolated and green *C. camphora* at the genus level. Compared with those of untreated plants, the application of the JZ-GX1 strain decreased the relative abundances of *Rozellomycota*, *Nectriaceae*, *Agaricomycetes*, *Neocosmospora*, *Paraphoma*, and *Plectosphaerella* and significantly increased those of *Mortierella*, *Saitozyma*, *Penicillium*, and *Trichoderma* (Figure 9B). These microbes may be an important feature of the ability of the JZ-GX1 strain to alleviate *C. camphora* etiolation.

## Discussion

### *Rahnella aquatilis* JZ-GX1 reduces oxidative damage in *Cinnamomum camphora* under alkali stress by activating the antioxidant system

Under normal physiological conditions, ROS in plants are in a dynamic balance of being continuously produced and scavenged, but the exposure of plants to biotic or abiotic stress destroys this balance and increases the level of ROS (Pflugmacher et al., 2010; Wan et al., 2015; Wu et al., 2021). These increases in ROS (including H_2_O_2_ and O_2_^−^) can lead to cell membrane damage and EL and subsequently to membrane lipid peroxidation, and the degree of membrane lipid peroxidation can be reflected by the MDA content (Guo et al., 2019). In this experiment, we found that iron deficiency led to increases in the H_2_O_2_ and O_2_^−^ contents in *C. camphora* leaves, which was consistent with the results obtained by Zhou C. et al. (2017) and Song et al. (2018) for peanut and chrysanthemum.

Superoxide dismutase, POD, and CAT are important active oxygen-scavenging enzymes in plants that can reduce the damage caused by membrane lipid peroxidation (Qin et al., 2018). Superoxide dismutase can catalyse the disproportionation of the superoxide anion to H_2_O_2_, and H_2_O_2_ is then decomposed into H_2_O by CAT and APX (Wang et al., 2012). Peroxidase, an enzyme that is widely found in plants, contains iron in a porphyrin ring and has limited specificity. This enzyme can catalyse the decomposition of intracellular peroxidation products and plays an important role in maintaining the normal physiological function of leaves (Nounjan et al., 2021). Iron is an important cofactor in the synthesis of many enzymes. Once iron deficiency leads to decreased POD and CAT activities, plant cells accumulate more H_2_O_2_ (Hassan et al., 2020). In this study, the application of JZ-GX1 increased the activities of four antioxidant enzymes, reduced the production of ROS (H_2_O_2_ and O_2_^−^) and the accumulation of MDA, and effectively alleviated the oxidative stress caused by iron deficiency. In addition, due to the redox characteristics of iron ions, bioavailable Fe^2+^ is easily oxidized, and the application of JZ-GX1 improved the antioxidant capacity of trees and stabilized the transport of Fe^2+^ in plants to promote the transfer of Fe^2+^ from roots to stems and leaves compared with that observed in the CK treatment.

### Desferrioxamine secreted by *Rahnella aquatilis* JZ-GX1 promotes iron absorption by *Cinnamomum camphora*

Plant growth-promoting rhizobacteria play important roles in plant growth and development and disease control and help plants cope with various abiotic stresses (such as salt, heavy metal, and drought stresses; Qin et al., 2018; Guo et al., 2020; Hassan et al., 2020). To date, most studies have focused on the secretion of active substances *in vitro* and the host responses of microorganisms, but no in-depth study has investigated how microorganisms function in complex soil environments. In this study, based on whole genome prediction and UPLC-MS spectrometric analysis, it was shown that JZ-GX1 strain could secrete DFO, and the abundance of DFO in inoculated soil was 21-fold higher than that in uninoculated soil. Desferrioxamine was the first siderophore isolated from *Streptomyces*, is the most effective iron fertilizer known thus far (Pierwola et al., 2004; Liu et al., 2019) and has been widely used as the iron source of peanut, cucumber, tobacco, wheat and other plants (Yehuda et al., 2003, 2012; Fernandez et al., 2004). Desferrioxamine secreted by JZ-GX1 strain may contribute to the iron absorption of *C. camphora*.

### Iron deficiency chlorosis of *Cinnamomum camphora* may be related to the relative abundance of soil pathogenic fungi

For a long time, the physiological etiolation of *C. camphora* was considered to be related to high pH and low available iron content in soil, but the microbial flora of etiolating plant soil was ignored. The plant root microbiome is considered the second genome of plants and plays an important role in plant growth and development (Wozniak and Galazka, 2019; Cao et al., 2020). We hypothesize that the physiological etiolation of *C. camphora* may be related to the relative abundance of pathogenic fungi in its rhizosphere soil, and this hypothesis was proven by FUNGuild function prediction analysis. Iron is the core of many metabolic processes, particularly in alkaline environments, and is thus the focus of severe competition among organisms (Aznar et al., 2014). To survive, a pathogen competes for insoluble Fe^3+^ in soil; thus, the plant has less soluble iron, which results in iron deficiency chlorosis in leaves (McCully et al., 2019). In addition, a KEGG metabolic pathway analysis of soil metabolism revealed that some macrolides were highly enriched in the treatment group. JZ-GX1 promoted the secretion of antibiotics by other microorganisms, this effect could be a consequence of the change in the microbial abundance of specific taxa. Meanwhile, it cannot be excluded that DFO could also be produced by some of the microorganisms whose growth was stimulated by JZ-GX1. Therefore, exogenous beneficial microorganisms can adjust the community and structure of indigenous microorganisms; this results in a decline in pathogen abundance, plants no longer lack iron, and the physiological etiolation can be alleviated.

### *Rahnella aquatilis* JZ-GX1 regulates specific soil fungal communities by secreting DFO

Studies conducted in recent years have found that siderophores can promote the growth of some unculturable microorganisms and change the microbial community (Xavier, 2011). In view of the high abundance of DFO obtained with the JZ-GX1 treatment in this study, we speculated that the rapid and stable secretion of DFO by JZ-GX1 into the environment provides not only abundant absorbable iron but also excessive siderophores, resulting in relatively high iron concentration niches, which are beneficial to the growth of other microorganisms using homologous siderophores. Studies have shown that some fungi of *Mortierella* and *Trichoderma* can secrete hydroxamic acid siderophores, and DFO belongs to this type of siderophore (Zhao et al., 2014). Therefore, we hypothesize that the observed increases in the *Mortierella* and *Trichoderma* populations may depend on the increase in DFO abundance found in the group inoculated with the JZ-GX1 strain. In addition, the excessive secretion of DFO-bound Fe^3+^ by the JZ-GX1 strain will bring excessive redox pressure to the environment. This type of siderophore can be recognized, absorbed and utilized by only the producer’s own specific receptors on the cell membrane and not by other microorganisms, thus inhibiting the growth of some microorganisms that cannot use it (Maindad et al., 2014; Yu et al., 2017). This mechanism may explain how the JZ-GX1 strain promotes plant development as a provider of iron in the rhizosphere. Therefore, in the future, the composition of fungi and bacteria in soil can be changed by adding biological fertilizer to the plant rhizosphere, and this strategy might provide a feasible biological method for the treatment of *C. camphora* etiolation from a microbial community perspective.

## Conclusion

**Figure 10:**
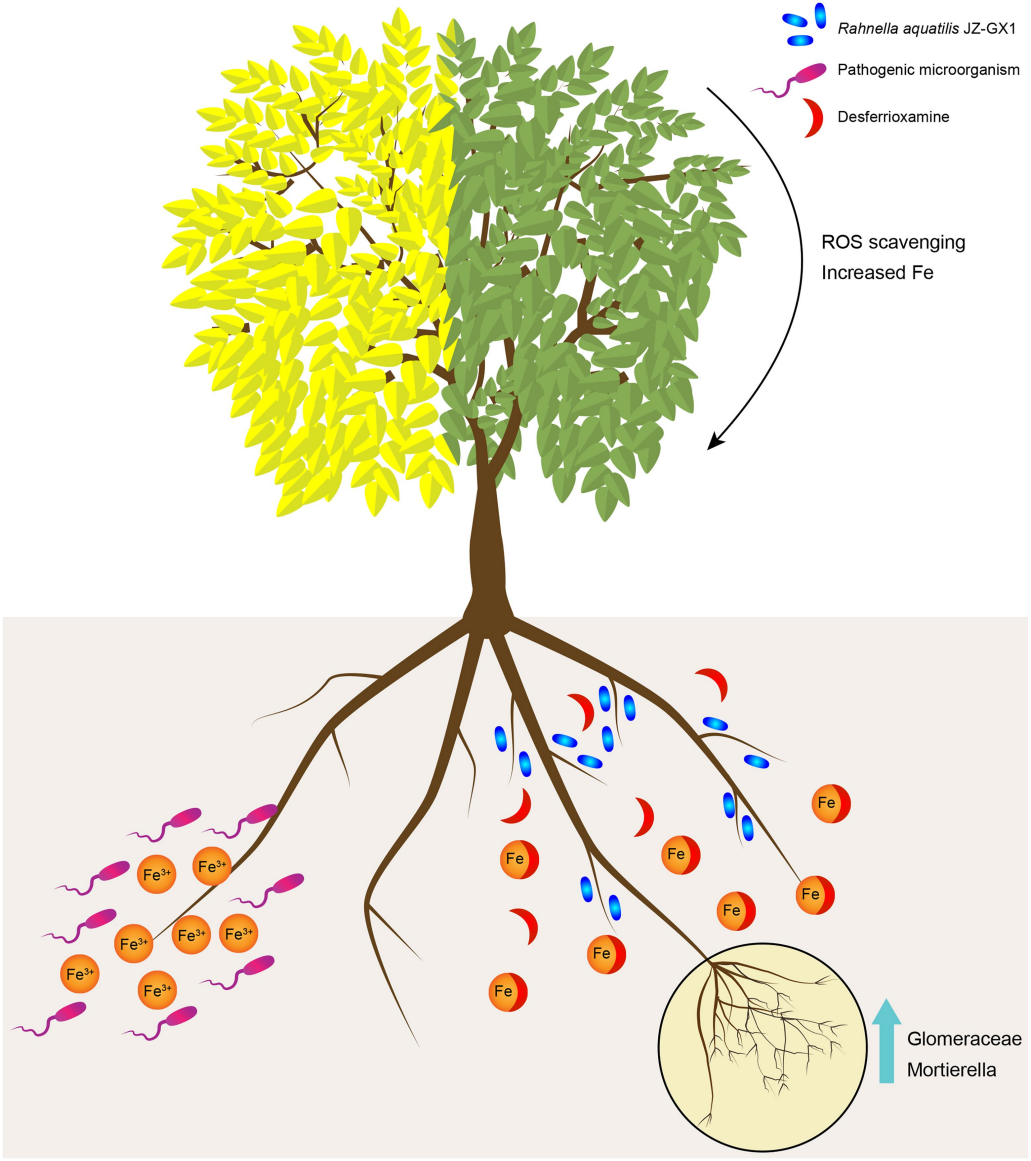
Putative model of the mechanism through which *Rahnella aquatilis* JZ-GX1 alleviates the iron deficiency-induced etiolation of *Cinnamomum camphora* by reprogramming the rhizosphere microbial community and increasing the content of DFO in soil. The JZ-GX1 strain secrete DFO to chelate insoluble Fe^3+^, then the Fe^3+^-DFO complex directly provides an iron source for iron-deficient *C. camphora*. At the same time, the removal of Fe^3+^ may limit the growth of pathogens and increase the population of *Glomeromycota* and *Mortierella*. The mycorrhizae formed by these microorganisms alleviate the etiolation of *C. camphora* caused by iron deficiency.

Plant growth-promoting rhizobacteria have been widely shown to promote plant growth and stress resistance. In this study, the exogenous application of JZ-GX1 increased the contents of chlorophyll, active iron and the activities of antioxidant enzymes in leaves. After a comprehensive analysis of the microbial community structure and metabolic response of *C. camphora* rhizosphere soil after inoculation, this study proposed the possible mechanism of interactions among *R. aquatilis* JZ-GX1, native microflora and plants (Figure 10). After entering the low-iron rhizosphere soil, the JZ-GX1 strain first secretes DFO to chelate insoluble Fe^3+^ for its own needs. The Fe^3+^-DFO complex directly provides an iron source for iron-deficient *C. camphora*. On the other hand, the removal of Fe^3+^ may limit the growth of pathogens and increase the abundances of some specific microorganisms (such as *Glomeromycota* and *Mortierella*). The mycorrhizae formed by these microorganisms indirectly promote the ability of plants to absorb iron and ultimately alleviate the etiolation of *C. camphora* caused by iron deficiency.

## Data availability statement

The datasets presented in this study can be found in online repositories. The names of the repository/repositories and accession number(s) can be found at: https://www.ncbi.nlm.nih.gov/, PRJNA733870.

## Author contributions

W-LK completed the data analysis and the first draft of the paper. W-LK, P-SL, and YZ were the finishers of the experimental research. Y-HW and L-XL participated in the experimental result analysis. X-QW directed experimental design, data analysis, and paper writing and revision. All authors contributed to the article and approved the submitted version.

## Funding

This work was supported by the National Key Research and Development Program of China (2017YFD0600104), the Priority Academic Program Development of the Jiangsu Higher Education Institutions (PAPD), and the Postgraduate Research & Practice Innovation Program of Jiangsu Province (KYCX20_0872).

## Conflict of interest

The authors declare that the research was conducted in the absence of any commercial or financial relationships that could be construed as a potential conflict of interest.

## Publisher’s note

All claims expressed in this article are solely those of the authors and do not necessarily represent those of their affiliated organizations, or those of the publisher, the editors and the reviewers. Any product that may be evaluated in this article, or claim that may be made by its manufacturer, is not guaranteed or endorsed by the publisher.

## Supplementary material

The Supplementary material for this article can be found online at: https://www.frontiersin.org/articles/10.3389/fpls.2022.960750/full#supplementary-material

## Footnotes

^1^ https://antismash.secondarymetabolites.org/

^2^ http://www.genome.jp/kegg/

^3^ www.majorbio.com
