# Supplementary material for: Rahnella aquatilis JZ-GX1 alleviates iron deficiency chlorosis in Cinnamomum camphora by secreting desferrioxamine and reshaping the soil fungal community
Source: Front Plant Sci. 2022 Sep 15;13:960750. doi: 10.3389/fpls.2022.960750 (PMC9520127; doi:10.3389/fpls.2022.960750)
Supplement: Supplementary file 1 [file Data_Sheet_1.DOCX]

Table S1 Primers for detecting siderophore synthesis gene expression of *R. aquatilis*

| Gene name | Primer (5'-3') | Function |
| --- | --- | --- |
| *dfoA* | GGCAGGCGTGACGATATG | lysine/ornithine N-monooxygenase |
|  | GTGCGGCGTGATGAAGATA |  |
| *dfoC* | CATCCTTCATTTCACTAC | siderophore biosynthesis protein |
|  | CTTCTAACCGTCTTACTA |  |
| *dfoJ* | CAGTAACACCATCAGCACAT | pyridoxal-dependent decarboxylase |
|  | GCCGTAAAGCGAGCATTA |  |
| *dfoS* | AAGATGGCGATGGAGTTG | siderophore MFS transporter |
|  | CAAAGTGCGTAACGGTTT |  |
| *atpD* | CAAAGTGCGTAACGGTTT | Reference gene |
|  | CGTGATAGAAGTCGTTACCCTC |  |


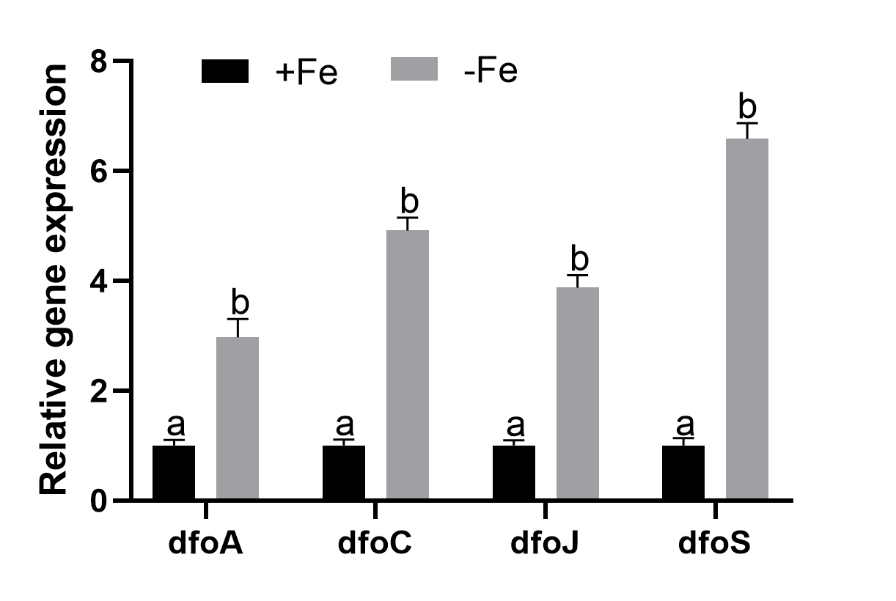


Figure S1. Relative expression of siderophore synthesis gene in JZ-GX1 strain under low iron (-Fe) and sufficient iron (+Fe) conditions.

A

B

Figure S2. Mass spectrum of DFO standard (A) and DFO in JZ-GX1 strain (B).
